# Infection of Powdery Mildew Reduces the Fitness of Grain Aphids (*Sitobion avenae*) Through Restricted Nutrition and Induced Defense Response in Wheat

**DOI:** 10.3389/fpls.2018.00778

**Published:** 2018-06-18

**Authors:** Zhi-Wei Kang, Fang-Hua Liu, Xiao-Ling Tan, Zhan-Feng Zhang, Jing-Yun Zhu, Hong-Gang Tian, Tong-Xian Liu

**Affiliations:** ^1^State Key Laboratory of Crop Stress Biology for Arid Areas, and Key Laboratory of Northwest Loess Plateau Crop Pest Management of Ministry of Agriculture, Northwest A&F University, Yangling, China; ^2^State Key Laboratory of Integrated Management of Pest and Rodents, Institute of Zoology, Chinese Academy of Sciences, Beijing, China; ^3^State Key Laboratory for Biology of Plant Diseases and Insect Pests, Institute of Plant Protection, Chinese Academy of Agricultural Sciences, Beijing, China

**Keywords:** *Blumeria graminis*, *Sitobion avenae*, nutrition, phytochemicals, *Aphidius gifuensis*

## Abstract

In natural ecological systems, plants are often simultaneously attacked by both insects and pathogens, which can affect each other’s performance and the interactions can be extended to higher trophic levels, such as parasitoids. The English grain aphid (*Sitobion avenae*) and powdery mildew (*Blumeria graminis* f. sp. *tritici*) are two common antagonists that pose a serious threat to wheat production. Numerous studies have investigated the effect of a single factor (insect or pathogen) on wheat production. However, investigation on the interactions among insect pests, pathogens, and parasitoids within the wheat crop system are rare. Furthermore, the influence of the fungicide, propiconazole, has been found to imitate the natural ecosystem. Therefore, this study investigated the effects of *B. graminis* on the biological performance of grain aphids and the orientation behavior of its endoparasitic wasp *Aphidius gifuensis* in the wheat system. Our findings indicated that *B. graminis* infection suppressed the feeding behavior, adult and nymph weight, and fecundity and prolonged the developmental time of *S. avenae*. We found that wheat host plants had decreased proportions of essential amino acids and higher content of sucrose following aggravated *B. graminis* infection. The contents of Pro and Gln increased in the wheat plant tissues after *B. graminis* infection. In addition, *B. graminis* infection elicited immune responses in wheat: increase in the expression of defense genes, content of total phenolic compounds, and activity of three related antioxidant enzymes. Moreover, co-infection of *B. graminis and S. avenae* increased the attraction to *A. gifuensis* compare to that after infestation with aphids alone. In conclusion, our results indicated that *B. graminis* infection adversely affected the performance of *S. avenae* in wheat through restricted nutrition and induced defense response. Furthermore, the preference of parasitoids in such an interactive environment might provide an important basis for pest management control.

## Introduction

In nature, herbivorous insects are known to attack host plants along with a variety of other species, including both pathogens and natural enemies (Stout et al., 2006; Tack et al., 2013; Franco et al., 2017). Plants serve as shared hosts allowing interactions among these species. Insects and pathogens have multiple interactions that might result in altered host plant quality and plant defense responses (Biere and Tack, 2013; Mauck et al., 2014). In some case, herbivory by an insect pest primes the host plant immune response, making the plant resistant to future pathogen infection. For example, an infestation of *Sitobion avenae* elicits plant defense responses, which then inhibit subsequent infection of *Fusarium graminearum* (De Zutter et al., 2017). Similarly, feeding by the white-backed planthopper, *Sogatella furcifera*, induced host plant resistance to rice blast caused by *Magnaporthe grisea* (Kanno and Fujita, 2003; Kanno et al., 2005). Another study suggested physiological changes in cotton seedlings caused by previous exposure to spider mites, *Tetranychus urticae*, which reduced the probability of infection and severity of symptoms caused by the wilt fungus *Verticillium dahlia* (Karban et al., 1987).

However, when pathogen infection precedes insect pest infestation, physiological changes in host plants have been found to adversely affect the biology of herbivorous insects. For instance, when the leaf beetle *Gastrophysa viridula* was fed rust-infected leaves from *Rumex obtusifolius*, it laid around 55% fewer eggs than when it was fed tissues from healthy plants (Hatcher et al., 2010). Further, both necrotrophic fungus *Phoma destructiva* (Plowr) and biotrophic fungus *Puccinia punctiformis* infection adversely affected larval development and increased larval and pupal mortality of *Cassida rubiginosa* (Kluth et al., 2002; Kruess, 2002). Conversely, pathogen infections occasionally enhance the performance of their co-host insects (Stout et al., 2006; Tack et al., 2013). Whiteflies *Bemisia tabaci* Middle East-Asia Minor 1 (MEAM1) fed on begomovirus-infected plants showed substantial increases in longevity and fecundity compared to whiteflies fed uninfected plants; however, the indigenous ASIA II 3 whitefly (formerly referred to as “ZHJ1 biotype”) did not show reproductive benefits when fed virus-infected plants (Jiu et al., 2007; Guo et al., 2012). In another case, researchers found that *Rhopalosiphum padi* underwent a 25% population size increased when reared on wheat infected with the *Barley Yellow Dwarf Virus* compared to a population reared on non-infected wheat (dos Santos et al., 2016).

Pathogen infection of host plants can also influence the preference and performance of natural enemies of their co-host pests. For example, the mass of the parasitoid wasp, *Cotesia glomerata* emerged from caterpillars (*Pieris brassicae*) reared on cabbage (*Brassica rapa*) with powdery mildew (*Erysiphe cruciferarum*) was significantly less than when parasitoids were generated from a non-infected cabbage system-even though the mass of the caterpillars themselves remained unchanged between the treatments (Ponzio et al., 2014). In addition, infection of *E. cruciferarum* significantly decreased the emission of host plant volatiles and reduced the attraction of *C. glomerata.* Conversely, *Xanthomonas oryzae* pv. *oryzae* (Xoo) infection promoted the emission of host plant volatiles and enhanced the preference of its co-host insect *Nilaparvata lugens*’ natural enemy, *Cyrtorhinus lividipennis* (Sun et al., 2016). Taken together, these studies have revealed that the nature of pathogen-insect-plant interactions depends on the species involved.

Phloem-feeding herbivores and biotrophic pathogens are generally found to primarily induce the salicylic acid (SA) signaling pathway, whereas leaf-chewing herbivores and necrotrophic pathogens usually trigger the jasmonic acid (JA) and ethylene (ET) signaling pathways (Glazebrook, 2005; Pieterse and Dicke, 2007; Pieterse et al., 2012). These different phytohormones have been shown to be also involved in the induction of plant volatiles (Van Poecke and Dicke, 2004; Wei et al., 2014). However, cross-talk can occur between these pathways, where induction of one pathway can have positive or negative regulatory effects on other pathways, and this is particularly the case between the SA and JA pathways (Koornneef and Pieterse, 2008; Spoel and Dong, 2008; Diezel et al., 2009; Pieterse et al., 2012; Thaler et al., 2012; Wei et al., 2014; Bonnet et al., 2017). However, in-depth analyses of the potential role of plant nutrition in this process are still lacking (Ponzio et al., 2017).

The English grain aphid, *Sitobion avenae* (Fabricius), and powdery mildew, *Blumeria graminis* f. sp. *tritici* (mildew), are worldwide crop antagonists causing in significant losses of wheat yield and commonly co-existing on wheat plants (Hu et al., 2015). Previously, the interactions of *S. avenae* and mildew with wheat have been documented independently. In this study, we investigated the three-way interactions among *S. avenae* and *B. graminis*, and their wheat host plant, *Triticum aestivum*. In particular, we investigated how the presence of mildew on wheat affects (1) the performance of *S. avenae*; (2) amino acids and soluble sugar contents in wheat; (3) transcript levels of genes associated with the SA pathway signaling relevant genes in wheat (*S. avenae*: phloem-feeding herbivore and mildew: biotrophic pathogen); and (4) the induction of defensive total phenolic and antioxidant enzymes; (5) the attraction of *S. avenae*’s main natural enemy, the parasitic wasp *Aphidius gifuensis*. To our knowledge, this is the first in-depth analysis of the nutritional composition and defense response in wheat plants during multiple interactions within this plant-insect-pathogen system.

## Materials and Methods

### Organisms Used in the Study

Seeds of winter wheat, *T. aestivum* (var. “XiNong 979”) were surface sterilized (1 min in distilled water) and individually germinated in pots (250 ml) containing a 3:1 mixture of peat moss (Pindstrup Mosebrug A/S; Ryomgaard, Denmark) and perlite, grown in a climate room at 23 ± 1°C, 60 ± 5% RH, and a L:D = 16:8 h photo regime. Seedlings were used in the experiment when they were 6 days old.

The culture of grain aphids (*S. avenae*) originated from individuals collected from wheat plants at a nature conservation site (Yangling, China) in June 2014. One aphid was reared for multiple generations on 6- to 20-day-old wheat plants (var. “XiNong 979”) in a climate-controlled room similar to the wheat-growing chamber described above. To obtain first-instar nymphs that were used in the experiment, we inoculated several 6- to 10-day-old wheat plants with apterous adult aphids. After 24 h, all adult aphids were carefully removed from the wheat, and the produced offspring were collected for use.

*Blumeria graminis* f. sp. *tritici* are obligate parasitic fungi that utilize host plant nutrients, reduce photosynthesis, impair growth, and reduce yields (Agrios, 1997). The biotrophic fungus *B. graminis* was obtained from the Key Laboratory of Northwest Loess Plateau Crop Pest Management of Ministry of Agriculture, Northwest A&F University (Shaanxi, China) and cultivated on wheat plants (XN “979”) under the laboratory conditions same as those for the wheat. For the inoculation, spores of *B. graminis* were collected and adjusted to a concentration 1 × 10^5^ spores/ml. The first and second leaves of wheat were sprayed with 100 μL of freshly prepared spore suspension or sterile water. To ensure spore germination, all treatments were covered with a transparent plastic bags to keep a high humidity for 24 h.

Propiconazole is a triazole fungicide and one of the most widely used fungicides in controlling powdery mildew on enormous plants in China. Furthermore, mildew (*B. graminis*) used in this work is susceptible to Propiconazole. Within 2 days, Propiconazole effectively controls the severity of mildew and lasts for 10–15 days. Propiconazole is bought from Syngenta AG (Dosage form: emulsifiable concentrates; active ingredient content: 250 g/L).

Aphid parasitic wasp, *A. gifuensis* Walker, was collected from a greenhouse in Yangling, Shaanxi, China. All mummified aphids were cultured in culture dishes in a climate room until emergence. About 40–50 adult wasps in a 1: 1 male-to-female sex ratio were released in a cage (40 × 40 × 40 cm) containing cabbages heavily infested with *Myzus persicae*. In the Y-tube olfactometer tests, females were between 3 and 5 days old. During experiments, females were collected and maintained individually in microcentrifuge tubes.

### Experimental Design

**FIGURE 1:**
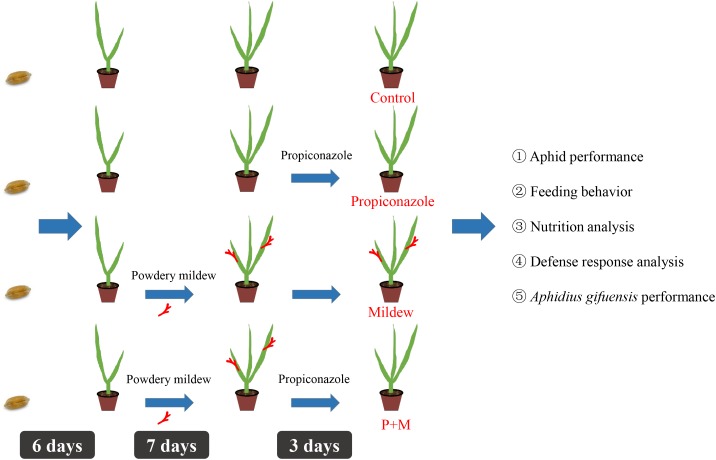
Timeline indicating important time points in the experiment investigating how the presence of Mildew on wheat affects the performance of *S. avenae* and its natural enemy – a parasitic wasp *Aphidius gifuensis*.

To investigate the possible effects of mildew infection on *S. avenae* and how the presence of mildew on wheat affects the performances of *S. avenae* and of its natural enemy – a parasitic wasp *A. gifuensis*, we assayed the plant response to mildew infection by using nutrient and defense analyses. To imitate the natural ecosystem, we also investigated the influence of propiconazole (fungicide). A detailed timeline of the experimental process is shown in **Figure 1**.

### Assessment of Aphid Performance

The performance of *S. avenae* under different treatments was evaluated using life table parameters. One newborn apterous *S. avenae* nymph was reared on either healthy or mildew-infested 16-day wheat, which was covered with a ventilated transparent plastic cylinder (9 cm diameter and 7 cm height). For each treatment, 25 replicates were used. Data on the development and fecundity of individual aphids were recorded every 12 h. Newborn nymphs were removed and weighed on a microbalance (resolution 0.001 mg; Sartorius MSA 3.6P-000-DM; Gottingen, Germany; 40 replicates). The period of each aphid from birth to the first production of offspring was expressed as Td, and the number of nymphs produced by each aphid for a period equal to the corresponding Td was regarded as Md. The intrinsic rate of increase (rm) for each aphid was estimated using the following equation: rm = 0.738 × (lnMd)/Td (Wyatt and White, 1977). At the end of this experiment, each adult aphid was collected and weighed on a microbalance.

### Aphid Feeding Behaviors

The probing and feeding behaviors of aphids sessile on healthy versus mildew-infested wheat were also monitored using the electrical penetration graph (EPG) technique (Kang et al., 2018). Individual aphids were pre-starved for 60 min and placed centrally on the third leaf from the apical meristem. The whole aphid-plant system was placed in a Faraday cage in a climate-controlled room at a temperature of 25 ± 2°C. Each aphid was monitored continuously for 8 h during the daytime and at least 17–18 successful replicates for each treatment were obtained. Acquisition and analysis of data were performed using the Stylet+d software (W.F. Tjallingii, Wageningen, Netherlands), and the stylet waveform was classified according to Tjallingii (1978). All behavioral variables were processed using the MS Excel Workbook for automatic EPG data calculation, developed by Sarria et al. (2009).

### Analysis of Amino Acids and Soluble Sugars in Phloem Exudates

Phloem exudates were collected from the third leaves of each pre-treated wheat plants following the procedure described in Bezemer et al. (2005) and Cao et al. (2014) with minor modifications. The leaf about 2 cm from the stalk was removed using a sharp scalpel while keeping it immersed in 1 ml of 5 mM EDTA solution (pH 7.0) in 1.5 ml Eppendorf (EP) tube. Two leaves from the same treatment group were placed individually in 1.5 ml EP tubes and regarded as one replicate. The leaves were then placed for 4 h in a dark growth chamber at 24°C with ≥ 90% relative humidity. Leaves were removed from EP tubes, any droplets attached to the petioles were knocked off, and the samples centrifuged at 12000 *g* for 15 min. The supernatant was drawn into a 1-ml syringe, and then filtered through a 0.2-μm syringe filter. Phloem samples were frozen at -80°C immediately after collection until analysis. The amino acid and soluble sugar concentrations were analyzed using LC-MS (Thiele et al., 2008). Amino acid and soluble sugar concentrations in the phloem were corrected for the dry mass of the leaf from which the phloem exudate was collected (Kos et al., 2015).

### Analysis of Plant Defense Response

To assess the defense response of wheat, we analyzed the expression patterns of phenylalanine ammonia lyase (*PAL*, marker gene for the SA biosynthesis/signaling pathway), Lipoxygenase (*LOX*, marker gene for the JA biosynthesis/signaling pathway), peroxidase (*PEROX*, the plant’s redox state), NADPH oxidase (*NADPHOX*, the plant’s redox state), basic *PR1* proteins (indicating the SA-mediated defense response), *PR2* (β-1,3-glucanase), and *PR3* (class VII acidic chitinases) for each treatment (Su et al., 2015; De Zutter et al., 2017). In addition, total phenolic content and the total enzymatic activities of the total superoxide dismutase (T-SOD), peroxidase (POD), and catalase (CAT) of the third pre-treated wheat leaves were measured using the corresponding model substrates.

### RNA Extraction and Quantitative Real-Time PCR

TRIzol reagent (Takara Bio, Tokyo, Japan) was used to extract RNA from the third wheat leaves following manufacturer’s instructions. The RNA integrity was verified using 1% agarose gel electrophoresis and the quantity was assessed using a NanoDrop ND-2000 spectrophotometer. Next, 1 μg of high-quality RNA was used to synthesize the first strand complementary DNA (cDNA) by using a PrimeScript^®^ RT reagent Kit with gDNA Eraser (perfect Real Time; Takara, Tokyo, Japan) following manufacturer’s protocol. The synthesized cDNA was stored at -20°C. Gene primers used in this study were referenced from those published previously (Smith et al., 2010; De Zutter et al., 2017). The quantitative polymerase chain reaction (qPCR) was conducted as we described previously with three biological replicates (Kang et al., 2017). The Comparative 2^-ΔΔ^*^C^^t^* method was used to analyze the relative quantification.

### Enzyme Activity Assays

The total enzymatic activities of the total superoxide dismutase (T-SOD), peroxidase (POD) and catalase (CAT) of the third pre-treated wheat leaves were measured using the corresponding model substrates (three replicates per treatment) (Tawaha et al., 2007). Enzymes were extracted from 0.5 g fresh leaves in a porcelain mortar containing 4.5 ml of ice-cold PBS buffer (0.1 mol^-1^, pH = 7.4) (Cao et al., 2015). The homogenate was centrifuged at 3500 rpm for 10 min at 4°C and the resulting supernatant was used directly for spectrophotometric assays of T-SOD, POD, and CAT activities. The activities of T-SOD and POD were determined using commercial assay kits (Nanjing Jiancheng Bioengineering Institute, Jiangsu, China) according to manufacturer’s instructions. CAT activity was analyzed using the method of Slaughter and O’Brien (2000) with some modification. We added 20 μL of supernatant and 980 μL of 30% H_2_O_2_ to a cuvette and mixed them immediately. Next, the change of absorption of the mixture was measured using an ultraviolet spectrophotometer (NanoDrop, m; Thermo, Boston, MA, United States) at OD240 every 15 s for 11 times. The activity of CAT was defined as ΔOD240 per min per g fresh weight.

### Total Phenolic Content Quantification

The total phenolic content of the third leaves from the different treatments was also measured (Tawaha et al., 2007). For extraction, the fresh third wheat leaves were washed several times with distilled water and freeze-dried for 24 h through vacuum freeze drying, and then dried in a hot oven (60°C) for 1 h (Iamsaard et al., 2014) when the pre-treatment wheat age was 16, 19, and 25 days. Next, 20 mg dried leaves was weighed into a PE and extracted with 1 ml of 80% methanol at 37°C for 3 h in a shaking water bath. After cooling, the extract was centrifuged at 3500 *g* for 10 min, and the supernatant was recovered and stored at 4°C until use for the total phenolic content assay. The total phenolic content was estimated by using the Folin–Ciocalteu colorimetric method, based on the procedure of Singleton et al. (1999), using gallic acid as a standard phenolic compound. Briefly, 25 μl (two replicates) of the filtered extracts were mixed with 225 μl of distilled water and 1.25 ml of 0.2 N Folin–Ciocalteu reagent. After 5 min, 1 ml of saturated sodium carbonate (75 g/L) was added. The absorbance of the resulting solution was measured at 765 nm by using a microplate reader (Tecan Group Ltd., Switzerland) after incubation at 30°C in a dark room for 1.5 h with intermittent shaking. Quantitative measurements were performed, based on a standard calibration curve of six points: 0, 0.01, 0.0125, 0.015, 0.0175, 0.02 mg/ml of gallic acid in 80% methanol. The results of the TPCs were expressed as gallic acid equivalents (GAEs) in milligrams per gram of dry material.

### Assessment of Parasitoid Wasp Performance

The impact of mildew infection on the host plant preference of *A. gifuensis*, an endoparasitoid of *S. avenae*, was assessed using the Y-tube choice assays (Pan et al., 2014; Kang et al., 2018). All the wheat treatment groups were co-infected with 200 aphids (mixed-instars) for 72 h. Subsequently, the U-tube choice assay was conducted as described by Pan et al. (2014) with a slight modification. After every 10 individuals, we reversed the position of the arms. Further, after 20 individuals, all the glass vessels and Y-tubes were replaced with fresh materials, which had been rinsed with 95% ethanol and distilled water and dried in a hot oven (60°C).

### Statistical Analysis

The data of EPG, *S. avenae* performance, plant nutrition, gene expression, enzyme activity, and TPC were assessed using analysis of variance (ANOVA), and preference of *A. gifuensis* was evaluated using Student’s *t*-test in SPSS 21 (SPSS Inc, Chicago, IL, United States). SAS Version 9.1 was used to conduct multiple-dimensional principal component analysis (PCA) of different amino acid concentrations among different treatments.

## Results

### Performance of Aphids

**FIGURE 2:**
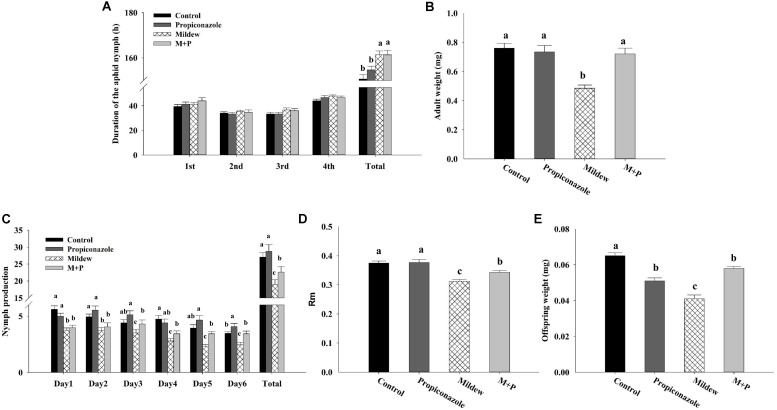
Performance of *S. avenae* on different plant species. **(A)** Duration of the aphid nymph (days for aphids to reach the adult stage). **(B)** Adult weight (mg). **(C)** Nymph production (the number of offspring laid per adult with 6 days). **(D)** Rm (the intrinsic rate of increase for each aphid). **(E)** Offspring weight (mg). Different letters above the bars indicate significant difference among different treatments and the error bars is ± SE bars (25 biological replicates, *P* < 0.05, Tukey’s HSD test).

Performance parameters of the grain aphid varied among treatments but were generally worse on Mildew and mildew plus propiconazole (M+P) treatments compared to that for other treatments (**Figure 2**). The adult weight was significantly lower on mildew-treated leaves than on other treatments (*F*_3,96_ = 15.773, *P* < 0.001). The offspring weight on control leaves was the highest among the treatments; it was the same on Propiconazole- and M+P-treated leaves, but was higher than that on Mildew-infected leaves (*F*_3,96_ = 26.383, *P* < 0.001). On Mildew- and M+P-treated leaves, aphids’ daily fecundity over the first 6 days and intrinsic rm were negatively affected compared with those measured on the two other treatments. In addition, an obvious difference was noted between the Mildew and M+P treatments for the aphids’ daily fecundity over the first 6 days and rm (*F*_3,96_ = 16.413, *P* < 0.001; *F*_3,96_ = 15.153, *P* < 0.001; **Figure 2D**). The development time of each instar remained unchanged for aphids regardless of host plant treatment, but the total development time was significantly lower for aphids on control leaves than on other treatments (*F*_3,96_ = 8.447, *P* < 0.001; **Figure 2A**). The numbers of nymphs of *S. avenae* on mildew and M+P-treated leaves were significantly lower than those on control and Propiconazole-treated leaves on the first 4 days of reproduction, but only the nymph numbers on mildew-treated leaves were negatively affected on days 5 and 6 (**Figure 2C**).

### Aphid Feeding Behaviors

The results revealed that nine EPG parameters were significantly different when the plant penetration behavior of *S. avenae* was monitored on the four differently treated wheat leaves (**Table 1**). The total aphid probing time was significantly lower (*F*_3,66_ = 3.837, *P* = 0.014) whereas the total duration of np was subsequently significantly longer on control wheat leaves than on the leaves of other treatments. The number and total duration of pathway phases increased on mildew-treated leaves compared to other treatments, and those on Propiconazole- and on M+P-treated leaves were the same which were higher than that on the control (*F*_3,66_ = 4.609, *P* = 0.006; *F*_3,66_ = 2.687, *P* = 0.054). On Mildew-treated wheat leaves, both the number and mean duration of pd were the highest among the four treatments (*F*_3,66_ = 4.475, *P* = 0.007; *F*_3,66_= 3.855, *P* = 0.014). The number and total duration of phloem salivation phase remained unchanged among treatments. The total duration of phloem ingestion significantly decreased on Mildew- and M+P-treated wheat leaves compared with that on the two other treatments (*F*_3,66_ = 4.358, *P* = 0.008), whereas the number of phloem ingestion phases remained unchanged. Time from the first probe to first sustained E2 (> 10 min) was not modified. Xylem ingestion occurred more often and was prolonged on Mildew- and M+P-treated wheat leaves than on control wheat leaves (*F*_3,66_ = 4.609, *P* = 0.006; *F*_3,66_ = 2.687, *P* = 0.054).

**Table 1:** Mean (± SE) sequential EPG variable values for the probing behavior of *Sitobion avenae* on four treatments wheats during an 8-h recording.

EPG Parameters	Control (*N* = 18)	Propiconazole (*N* = 18)	Mildew (*N* = 17)	M+P (*N* = 17)
Total probing time (Pr) (h)	6.41 ± 0.21*b*	7.04 ± 0.18*ab*	6.95 ± 0.16*ab*	7.24 ± 0.66*a*
Time from first probe to first sustained E2 (> 10 min) (h)	2.74 ± 0.36*a*	2.05 ± 0.36*a*	3.25 ± 0.54*a*	3.61 ± 0.54*a*
Total duration of E1 (h)	0.96 ± 0.26*a*	0.96 ± 0.26*a*	0.93 ± 0.19*a*	0.85 ± 0.26*a*
Total duration of E2 (h)	3.44 ± 0.42*a*	3.07 ± 0.50*b*	1.79 ± 0.31*c*	1.80 ± 0.39*c*
Mean duration of pd (s)	5.60 ± 0.74*b*	5.84 ± 0.74*b*	8.79 ± 0.93*a*	5.62 ± 0.74*b*
Total duration of C (h)	1.86 ± 0.14*b*	2.18 ± 0.20*ab*	2.66 ± 0.25*a*	2.22 ± 0.25*ab*
Total duration of np (h)	1.59 ± 0.21*a*	0.96 ± 0.18*ab*	1.05 ± 0.16*ab*	0.76 ± 0.18*b*
Duration of G (h)	0.85 ± 0.15*b*	1.06 ± 0.21*ab*	1.40 ± 0.26*ab*	1.94 ± 0.46*a*
Number of probes	12.43 ± 1.09*a*	11.59 ± 1.54*a*	14.41 ± 2.40*a*	10.43 ± 0.95*a*
Number of E1	4.52 ± 0.63*a*	4.57 ± 0.60*a*	5.60 ± 0.62*a*	4.43 ± 0.58*a*
Number of E2	4.11 ± 0.52*a*	4.00 ± 0.64*a*	3.93 ± 0.36*a*	3.27 ± 0.58*a*
Number of pd	90.00 ± 10.12*b*	88.27 ± 9.10*b*	132.73 ± 14.60*a*	81.33 ± 9.31*b*
Number of C	16.90 ± 0.95*b*	19.44 ± 1.81*ab*	25.93 ± 2.67*a*	19.73 ± 1.71*ab*
Number of G	2.60 ± 0.30*b*	3.46 ± 0.54*b*	6.18 ± 0.64*a*	6.29 ± 1.05*a*

*Means (± SE) in the same column with the same letter indicate that the means are not significantly different at P = 0.05 (Tukey’s HSD test). E1, salivation phase; E2, phloem sap ingestion; pd, C, pathway phase; G, xylem ingestion; np, non-probe phase. N, number of replicates.*

### Amino Acid and Soluble Sugars

**FIGURE 3:**
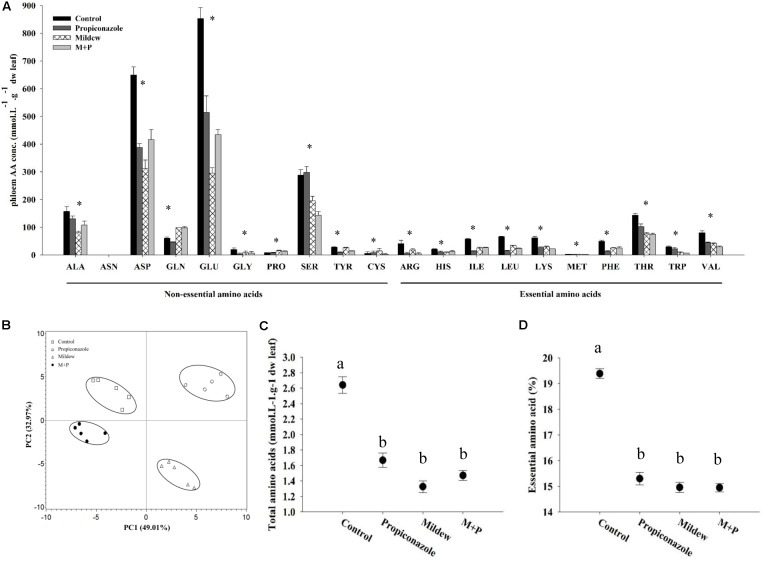
Concentrations of individual amino acid and total free amino acids, the proportion of essential amino acids, and amino acid composition profiles in the four plant phloem. **(A)** Concentrations of individual free amino acid (μM). **(B)** Amino acid composition profiles (PCA). **(C)** The concentration of total free amino acids (μM). **(D)** The proportion of essential amino acids (mol %). Asterisk or different letters above the error bars indicate significant differences among different treatments and the error bars is ± SE bars (five biological replicates, *P* < 0.05, Tukey’s HSD test).

A total of 19 amino acids were detected in the phloem of wheat leaves (**Figure 3A**). Among these 19 amino acids, the most abundant were Glu, Asp, Ser, Gln, Ala, and Thr. Except for Pro and Gln, mildew infection significantly decreased the concentrations of free amino acids (**Figure 3A**). The concentration of Pro and Gln in Mildew-treated leaves were significantly higher than those of control and Propiconazole-treated leaves (Pro: *F*_3,16_ = 10.794, *P* < 0.001; Gln: *F*_3,16_ = 79.381, *P* < 0.001; **Figure 3A**). A PCA with 81.98% variance of the data indicated that the variation of amino acids within sample replicates was smaller than that among different treatments (**Figure 3B**). The Mildew-infected wheat containing the lowest total amino acids (TAAs), and the healthy wheat had the highest TAAs (*F*_3,16_ = 27.587, *P* < 0.001; **Figure 3C**). The average ratio of essential amino acid to amino acids was significantly lower in Propiconazole-, Mildew-, and M+P-treated wheat leaves than in healthy wheat (*F*_3,16_ = 16.161, *P* < 0.001; **Figure 3D**).

**FIGURE 4:**
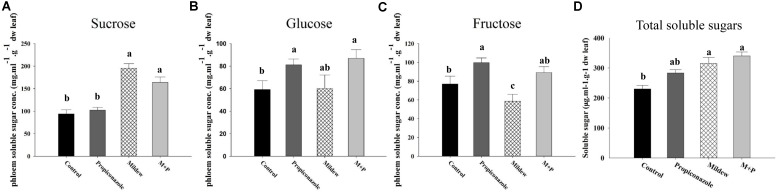
Simple carbohydrates among the four treatments. **(A)** Sucrose; **(B)** Glucose; **(C)** Fructose; **(D)** Total sugars. Different letters above the bars indicate significant difference among different treatments and the error bars is ± SE bars (five biological replicates, *P* < 0.05, Tukey’s HSD test).

The total soluble sugar in mildew and M+P-treated wheat was significantly higher than that of control and Propiconazole-treated wheat (*F*_3,16_ = 10.926, *P* < 0.001; **Figure 4D**). Source exhibited a similar pattern of total soluble sugar (*F*_3,16_ = 26.358, *P <* 0.001; **Figure 4A**) but the fructose in mildew-treated leaves was the lowest among all treatments (*F*_3,16_ = 6.645, *P* = 0.004; **Figure 4C**). No significant difference in glucose levels was noted among the four treatments (*F*_3,16_ = 2.829, *P* = 0.072; **Figure 4B**).

### Wheat Defense Response Following Treatment

**FIGURE 5:**
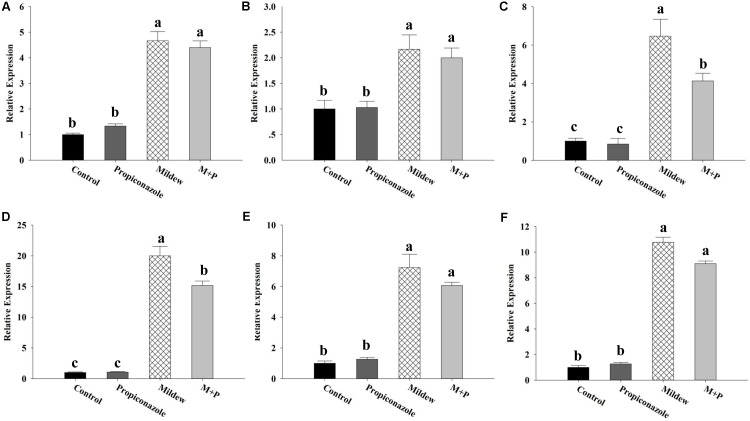
Expression profiles of defense-related genes in wheat of the four treatments. **(A)** *PAL*; **(B)** *PR1*; **(C)** *PR2*; **(D)**: *PR3*; **(E)** *PEROX*; **(F)** *NADPHOX.* Different letters above the bars indicate significant difference among different treatments and the error bars is ± SE bars (three biological replicates, *P* < 0.05, Tukey’s HSD test).

To investigate the defense response of wheat, we analyzed the defense gene expression, enzymatic activity, and total phenolic content among the four treatments. We found that all defense genes showed significant induction following mildew infection, in which gene expression was upregulated twofold to 20-fold (*PAL*: *F*_3,8_ = 74.112, *P* < 0.001, *PR1*: *F*_3,8_ = 7.355, *P* = 0.011, *PR2*: *F*_3,8_ = 48.271, *P* < 0.001, *PR3*: *F*_3,8_ = 131.923, *P* < 0.001, *PEROX*: *F*_3,8_ = 130.812, *P* < 0.001, *NADPHOX*: *F*_3,8_ = 495.834, *P* < 0.001; **Figure 5**). No significant difference in gene expression was observed between control and Propiconazole treatments and between mildew and M+P treatments for *PAL*, *PR1*, *PEROX*, and *NADPHOX*. Conversely, compared to mildew treatment, the application of Propiconazole decreased the expression of *PR2* and *PR3* (*PR2*: *P* = 0.003, *PR3*: *P* = 0.016; **Figures 5C,D**). *LOX* did not show any significant induction at the tested time points (**Supplementary Figure S1**).

**FIGURE 6:**
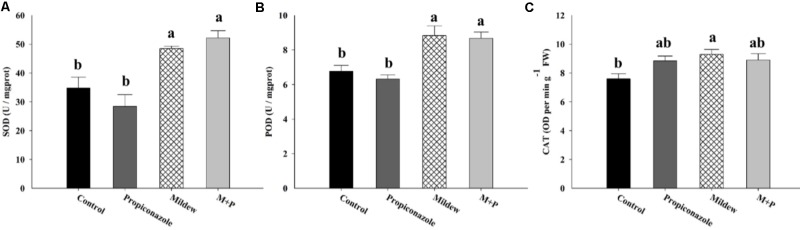
The activity of three antioxidant enzymes: SOD **(A)**, POD **(B)**, and CAT **(C)** among the four treatments. Different letters above the bars indicate significant difference among different treatments and the error bars is ± SE bars (three biological replicates, *P* < 0.05, Tukey’s HSD test).

The total enzymatic activities of T-SOD, POD, and CAT are considered representative of the plant detoxification system in response to damage (**Figure 6**). No significant differences were found in the activity of these three enzymes between Mildew- and M+P-treated leaves (**Figure 6B**). T-SOD and POD activities were significantly higher in Mildew and M+P treatments than in control and Propiconazole treatments (T-SOD: *F*_3,32_ = 13.018, *P* < 0.001; POD: *F*_3,32_ = 10.744, *P* < 0.001). In addition, no significance difference was noted between control and Propiconazole treatments and between mildew and M+P treatments. Further, CAT responses in wheat were only significantly different between mildew (higher) and control (lower) treatments (*F*_3,32_ = 4.046, *P* = 0.021; **Figure 6B**).

**FIGURE 7:**
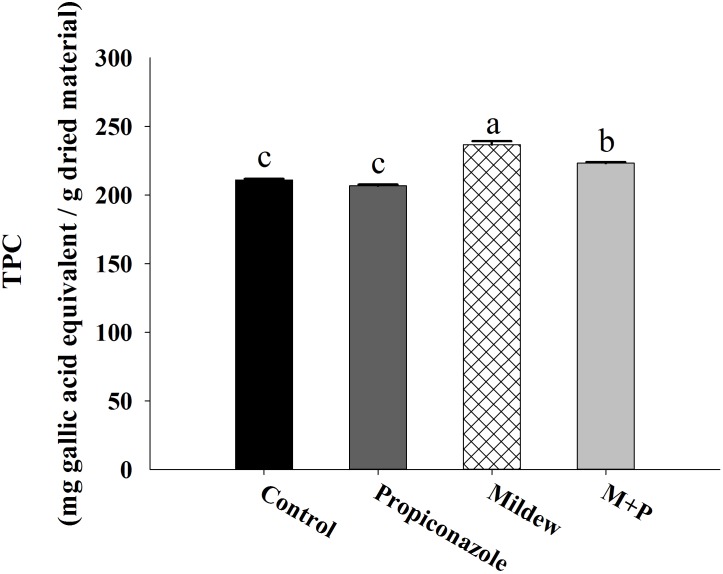
The total phenolic content in the wheat leaves of four treaments. Different letters above the bars indicate significant difference among different treatments and the error bars is ± SE bars (15 biological replicates, *P* < 0.05, Tukey’s HSD test).

The results of total phenolic contents of wheat leaves from the different treatments are shown in **Figure 7**. The calibration curve of gallic acid from this assay was *y* = 0.4369*x* + 0.0394, *R*^2^ = 0.9921 [*y* is the peal area and *x* is the concentration (mg/ml) of the marker compound]. Significant variation of total phenolic content in different wheat leaves was found according to the different treatments. The total phenolic content of mildew and M+P treatments were significantly higher than control and Prinopiconazole treatments, and the total phenolic content of Mildew-treated leaves was higher than that that in M+P treatment (*F*_3,56_ = 86.419, *P* < 0.001).

### Performance of Parasitoid Wasp

**FIGURE 8:**
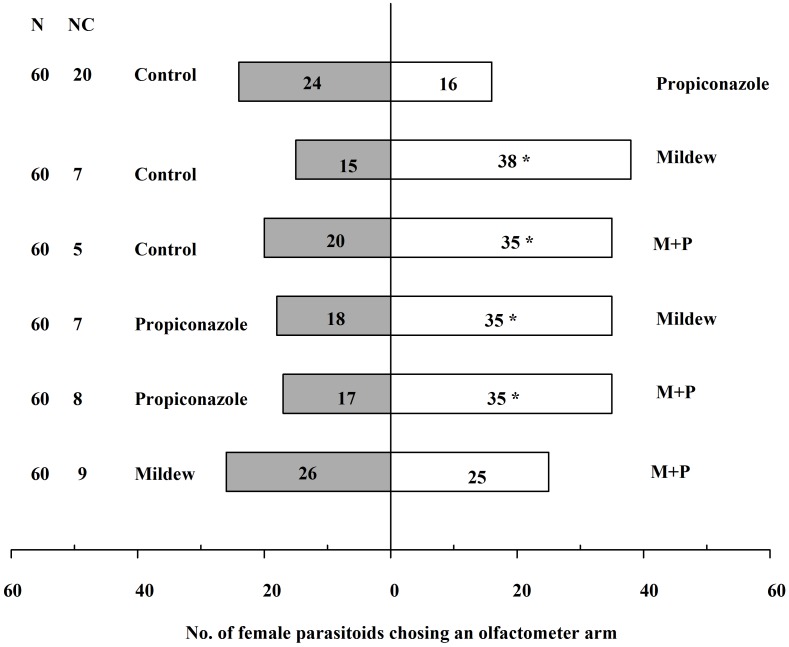
Preference of *A. gifuensis* among the different treatments.

Host plant preference of *A. gifuensis* was investigated using a Y-tube olfactometer choice assay. Wasps were exposed to volatile blends from the four treatment groups (results shown in **Figure 8**). *A. gifuensis* exhibited a higher preference for the Mildew-infected wheat regardless of the application of Propiconazole (control vs. Mildew: χ^2^ = 9.981, *P* = 0.002; control vs. M+P: χ^2^ = 4.091, *P* = 0.043; Propiconazole vs. Mildew: χ^2^ = 5.454, *P* = 0.020; Propiconazole vs. M+P: χ^2^ = 6.231, *P* = 0.013). *A. gifuensis* did not show significant preference between control and Propiconazole-treated wheat (χ^2^ = 1.600, *P* = 0.206), as well between Mildew and M+P (χ^2^ = 0.020, *P* = 0.889).

### A Schematic of Aphid-Pathogen-Plant Interaction

**FIGURE 9:**
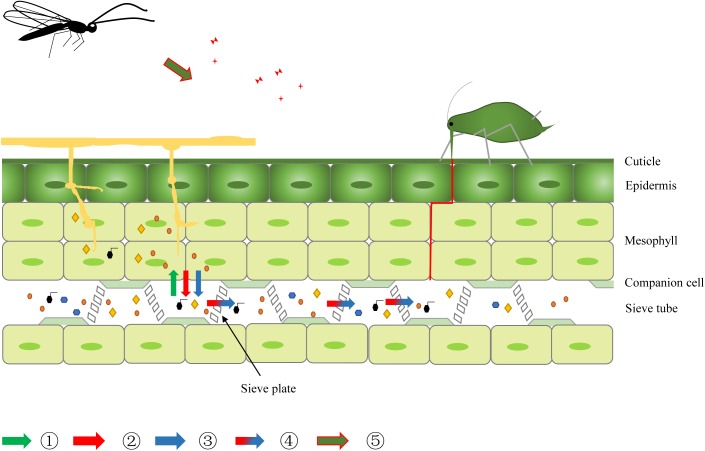
A schematic of aphid-pathogen-plant interaction.

Based on these results, we constructed a schematic of the multiple interactions of aphid-pathogen-plant (**Figure 9**). Green arrows indicate the competition of nutrition between aphid and pathogen (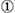); during pathogen infection, numerous toxins as well as other metabolites are released (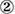); in addition to alterations in host plant metabolism, pathogen infection activates phytohormonal signaling pathways, which affect the defense and nutrient systems of plants (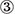); secondary metabolites of the pathogen and host plants are transported to the aphid feeding area and ingested by insects (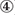); in addition, pathogen infection affects the volatile emissions of the shared host plants, which in turn changes the host-plant seeking behavior of the aphid’s natural enemy (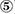).

## Discussion

Our results revealed not only the negative effects of mildew infection on the fitness of grain aphid *S. avenae*, but also the potential causes of this phenomenon.

In our study, we found that the adult and offspring weights of *S. avenae* reared on Mildew- and M+P-treated wheat were significantly lower than those of aphids reared on control and Propiconazole-treated wheat. No significant difference was noted in aphid adult weights between mildew and M+P treatment groups. In addition, the development period of aphids reared on mildew-infected wheat was significantly increased. These results were consistent with those of previous studies on the effects of *P. destructive* and *P. punctiformis* infestation on *Cassida rubiginosa* (Kluth et al., 2002; Kruess, 2002). This study showed that *P. destructive* and *P. punctiformis* infestation of host plants lengthened larval development time and decreased the weight of last-instar larvae and pupae of *C. rubiginosa* (Kluth et al., 2002; Kruess, 2002). This might be driven by the cross talk of plant defense related pathways mainly on JA and SA, where induction of one pathway can have positive or negative regulatory effects on other pathways (Koornneef and Pieterse, 2008; Spoel and Dong, 2008; Pieterse et al., 2012; Thaler et al., 2012; Wei et al., 2014; Bonnet et al., 2017). Similarly, another study showed that infection of *Chaetomium cochliodes* in *Cirsium arvense* significantly reduced the growth of *Mamestra brassicae* (Gange et al., 2012). In this study, we found that, when *S. avenae* were forced to feed on Mildew-infected plants, their overall reproduction was reduced. This is also consistent with the findings of previous study in another insect-pathogen-plant system: the population size of aphids reared on virus-infected Zinnia plants significantly reduced over time compared to that of aphids reared on healthy plants (Lowe and Strong, 1963). Similarly, Yang et al. (2013) found that the fecundity of the beet armyworm (*Spodoptera exigua*) decreased when the insects were fed a diet of mildewed rose leaves. Notably, in contrast with our results, a study showed that aphids (*Euceraphis betulae*) fed on the phloem of fungus-infected silver birch leaves were heavier, larger, and showed enhanced embryo development (Johnson et al., 2003). Moreover, the invasive MEAM1 whitefly fed on begomovirus-infected plants experienced substantial increase in longevity and fecundity compared to when they were fed uninfected plants, a benefit not observed for the indigenous ASIA II3 whitefly (Jiu et al., 2007). In-depth investigations revealed that MEAM1 whitefly fed on begomovirus-infected plants experienced enhanced fecundity via increased expression of an insulin-like peptide and increased vitellogenesis (Guo et al., 2012, 2014). In this study, the adult and offspring weight on Propiconazole- and M+P-treated wheat leaves was lower than that on healthy wheat leaves, whereas no significant difference was noted between Propiconazole- and M+P-treated wheat leaves. Propiconazole might be detrimental to *S. avenae* directly or it might induce resistance (Asalf et al., 2012), but the resistance was eliminated with the reduction in efficacy, and the resistance was weakened since the powdery mildew was controlled by the fungicide. Taken together, these findings suggest that pathogen infection of a host plant can either enhance or suppress the performance of resident herbivorous insects depending on the plant, pathogen, and insect species.

Our EPG results showed reduction in the duration of aphid’s phloem ingestion phases and prolonged the duration of aphid’s xylem ingestion phases on mildew-infected plants during the 8 h of recording. Under adverse feeding conditions such as feeding on resistant wheat cultivars, aphids showed more single-salivation period and shorter mean phloem ingestion duration than those fed susceptible cultivars (Tyree and Sperry, 1989; Carmo-Sousa et al., 2014). These results indicate that the infection of mildew infection increased the resistance of wheat to *S. avenae*, although *S. avenae* might prefer or avoid feeding on the pathogen-infected tissue. For example, the thistle tortoise beetle *Carduus thoermeri* and the true weevil *Trichosirocalus horridus* strongly selected non-infected leaf parts of rust-infected thistle leaves for feeding (Kok et al., 1996). In another study, in choice assays, the leaf beetle *Plagiodera versicolora* consumed less leaf area from the rust fungus *Melampsora alliifragilis* infected leaf halves as compared to uninfected halves of the same leaves (Simon and Hilker, 2005). To investigate the potential mechanism of the negative effects of powdery mildew on the grain aphid, we analyzed the changes in nutrition composition and defense responses of wheat infected with mildew.

Concerning the changes in nutrition composition of infected wheat, we found that the total amino acid and proportion of essential amino acids in Mildew-infected wheat were significantly lower than those in the healthy wheat. In a previous study, the essential amino acid composition of plants was fund to be the limiting factor of resident aphid populations (Guo et al., 2013; Russell et al., 2013; Liu et al., 2016; Züst and Agrawal, 2016). For example, a high proportion of essential amino acids evidently favored the population establishment of *Myzus persicae* on new plant species (Liu et al., 2016). Further, the fecundity and growth rate of the bird oat-cherry aphid, *R. padi*, was directly proportional to the FAA levels in its host plant (Weibull et al., 1990). Moreover, the performance of *Aphis glycines* was lower when the diet concentration of Val was reduced (Wille and Hartman, 2008). Non-essential amino acids also play critical roles in aphid-plant interactions (Srivastava et al., 1983). For example, Glu is imported into the resident symbiotic gut bacteria in aphids and used for the synthesis of essential amino acids (Liadouze et al., 1995). The reduction of Glu in the diet significantly reduced the performance of *A. glycines* (Wille and Hartman, 2008). Similarly, on artificial diets, Asn, Glu, and Pro were found to be essential for the growth and survival of *Acyrthosiphon pisum* (Srivastava et al., 1983). Tyr and Asp increased the weight of this aphid species, but did not prolong its survival. Another study showed that a lower level of Asn and Glu was found in rice plants with a higher resistance to *N. lugens* (Sogawa and Pathak, 1970). All these results indicate that the reduction of dietary essential amino acids negatively affects aphid fitness, which might be the reason for the poor performance of *S. avena*e on mildew-infected wheat in this study.

Unlike the major amino acids, Pro content was significantly increased in wheat tissues following mildew infection. In previous studies, the accumulation of free Pro has been found in various plants in response to abiotic and biotic stresses such as drought, high salinity, heavy metals, pathogen infection, and insect pest attack (Szabados and Savoure, 2010; Chen et al., 2011; Qamar et al., 2015). For example, Pro metabolism is involved in the ROS burst and the hypersensitive response triggered by an avirulent pathogen (Zeier, 2013). Moreover, leaf tissues treated with exogenous Pro solutions in millimolar concentrations elicited a series of resistance mechanisms including SA accumulation, ROS formation, and *PR* gene expression (Chen et al., 2011). Thus, the accumulation of Pro in mildew-infected wheat indicated that the wheat defensive mechanisms had been triggered. Based on the qPCR and enzyme activity data shown in **Figures 4**, **5**, we found that mildew infection significantly induced defense responses in wheat, which was consistent with the findings of previous proteomic and transcriptomic investigations on the effects of mildew infection of wheat (Xin et al., 2012; Tayeh et al., 2015; Fu et al., 2016; Li et al., 2017). In these studies, numerous genes involved in phytohormone metabolism and defensive signaling pathways were upregulated in response to mildew infection, including SA, abscisic acid, gibberellic acid, ET, Auxin, and cytokinin. The triggering of these phytohormone metabolism and signaling pathways induced the activity of plant oxidative enzymes and the accumulation defensive metabolites, which are correlated with host plant resistance to aphid herbivory. In the literature, higher levels of POD were found in resistant plants than in susceptible ones following aphid feeding (Cao et al., 2015). The high efficiency of SOD and CAT was essential for pea seedlings to overcome pea aphid-induced oxidative stress (Mai et al., 2013; Morkunas et al., 2016). Furthermore, in *R. padi*, a higher concentration of total phenol in resistant cultivars was thought to be positively correlated with higher mortality of resident aphids (Leszczynski et al., 2011). These results support to our proposition that mildew infection provokes earlier, similar, and/or enhanced typical sensitive plant responses against *S. avenae*. Moreover, plant changes and secondary metabolites produced by pathogens can adversely influence insects, leading to altered preferences and performances, such as reproduction, population enlargement and survival (Mauck et al., 2014).

In addition to host plant defense response, in this study, we also investigated how pathogen presence affects the preference of their co-host pest’s natural enemy. We found that wheat co-infected by mildew and *S. avenae* was more attractive to the aphid endoparasitoid *A. gifuensis*. Consistent with our results, Cardoza et al. (2003) found that wasps were more responsive to volatiles from plants infected with white mold compared with those from healthy ones when both types of plants were damaged by beet armyworms caterpillars. Further, the infection of cereal yellow dwarf virus (CYDV) increased the vulnerability of its vector to be parasitized by parasitoid wasp, *Aphidius colemani* (de Oliveira et al., 2014). However, Rostas and Hilker (2002) found that plants infected with only *Setosphaeria turcica*, which is a necrotrophic fungus, showed no attraction to two parasitic wasp species of *Spodoptera littoralis*. Conversely, compared with to plants infested with the larvae of *S. littoralis* only, the co-infection of *S. turcica* enhanced their preference. Furthermore, the presence of powdery mildew *Erysiphe cruciferarum* on the leaves of cultivated *Brassica* species significantly decreased the efficiency of the parasitoid *C. glomerata* against *P. brassicae* caterpillars (Ponzio et al., 2014). These dual responses of natural enemies relied on the type of released volatiles following pathogen infection. For example, volatile organic compounds emitted from the Xoo-infected rice were significantly higher than those from healthy rice plants (Sun et al., 2016). More interestingly, the co-infection of Xoo and *N. lugens* caused rice plants to emit more volatiles than the herbivore-infested plants alone. In contrast, compared that in healthy plants, quantitatively 41% less volatile emission was noted for mildew-infected plants. In addition, the volatile blends of insect-infested treatments occasionally differed from those in non-infested plants. These results suggested that pathogen infection affects the preference of the natural enemies of their co-host pests relying on the volatile emissions of host plants (Tack et al., 2013). In some cases, pathogen infection induces or increases plant volatile emissions containing the natural enemy attractive cues of their co-host pest. Conversely, pathogen infection occasionally decreases plant volatile emissions or induces the production of volatiles without hampering the attractive cues of natural enemy to their co-host pest or even containing the deterrents for aiding natural enemies. Thus, natural enemy attraction to host plants mediated by volatile odor cues depends on the pathogen, pests, and plant species in question (Hauser et al., 2013; Tack et al., 2013). Owing to technology limitation, we could not perform the volatile analyses.

## Conclusion

We provide valuable information regarding the impact of mildew on the performance of *S. avenae* and *A. gifuensis*. In addition, we showed that mildew infection reduced the fitness of *S. avenae* through restricted nutrition and induced defense response in wheat host plant. Moreover, fungal pathogen controlled by fungicides can prevent co-host insect development and has no effect on the host plant growth. If these findings could be applied under field conditions, they might have some implications for the integrated control of *S. avenae* and mildew in wheat. Propiconazole should be applied first to control mildew, and then *A. gifuensis* can be released to control *S. avenae*. Our findings not only broaden our knowledge on plant-pathogen-insect interactions but also might aid in the development of durable ways to integrate the management of plant pathogens and insect herbivores in agroecosystems.

## Author Contributions

Z-WK, F-HL, and T-XL conceived the ideas and designed the methodology. Z-WK and F-HL conducted the experiment and collected the data. Z-WK, F-HL, X-LT, Z-FZ, J-YZ, and H-GT analyzed the data. Z-WK, F-HL, and T-XL wrote the manuscript. All authors contributed critically to the drafts and gave final approval for the publication.

## Conflict of Interest Statement

The authors declare that the research was conducted in the absence of any commercial or financial relationships that could be construed as a potential conflict of interest.

## Supplementary Material

FIGURE S1 Expression profiles of *LOX* among the four treatments.
