# Supplementary figures and images for: Infection of Powdery Mildew Reduces the Fitness of Grain Aphids (Sitobion avenae) Through Restricted Nutrition and Induced Defense Response in Wheat
Source: Front Plant Sci. 2018 Jun 18;9:778. doi: 10.3389/fpls.2018.00778 (PMC6015903; doi:10.3389/fpls.2018.00778)

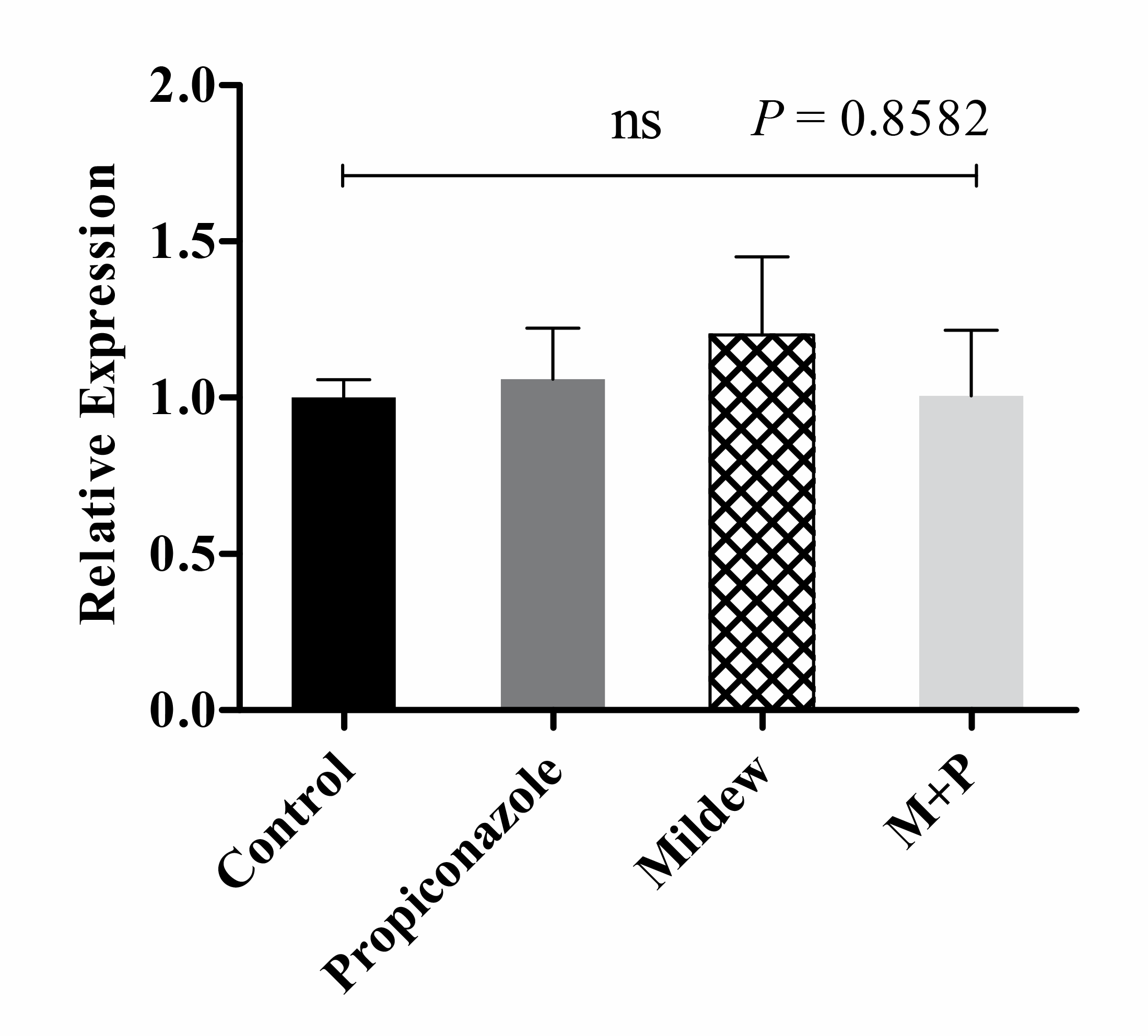

Supplement: FIGURE S1 — Expression profiles of LOX among the four treatments. [file Image_1.TIF]
